# Prognostic factors and cervical lymph node management in tongue squamous cell carcinoma: a retrospective cohort study

**DOI:** 10.1007/s00784-026-06754-6

**Published:** 2026-03-07

**Authors:** Mario Scheurer, Tobias Daut, Johannes Schulze, Michael Grunert, Alisa Schramm, Robin Kasper, Frank Wilde, Alexander Schramm, Majeed Rana, Marcel Ebeling, Andreas Sakkas

**Affiliations:** 1 Department of Oral and Maxillofacial Surgery, University Hospital Ulm, Ulm, 89081 Germany; 2 Department of Oral and Plastic Maxillofacial Surgery, Military Hospital Ulm, Ulm, 89081 Germany; 3 Department of Nuclear Medicine, Military Hospital Ulm, Ulm, 89081 Germany; 4 Department of Nuclear Medicine, University Hospital Ulm, Ulm, 89081 Germany; 5 Department of Otorhinolaryngology, Military Hospital Ulm, Ulm, 89081 Germany

**Keywords:** TSCC, [^18^F] FDG PET/CT, Lymphatic drainage, Neck dissection, Patient-specific medicine, Personalized oncology

## Abstract

**Objectives:**

The optimal management of cervical lymph nodes in tongue squamous cell carcinoma (TSCC) remains controversial, given the need to balance oncological safety with functional preservation. This study aimed to identify clinicopathological predictors of recurrence and survival to improve patient-specific strategies for neck management.

**Materials and methods:**

This retrospective study included 74 patients with histologically confirmed TSCC. All patients underwent standardized staging including [¹⁸F] FDG PET/CT. Predictor variables included clinical tumor size (cT), suspicious cervical nodes (cN) and number of metastatic lymph nodes (pN). Outcome parameters comprised overall survival (OS), recurrence-free survival (RFS), local recurrence, regional recurrence and contralateral nodal recurrence and disease-specific mortality.

**Results:**

The 1- and 5-year OS rates were 97% and 83%, respectively, and RFS rates were 80% and 61%. Perineural invasion (PNI) was significantly associated with inferior OS (*p* = 0.03) and RFS (*p* = 0.021). The number of metastatic lymph nodes independently predicted mortality (OR = 1.5; 95% CI: 1.08–2.16; *p* = 0.018). Each additional suspicious node on [^18^F] FDG PET/CT increased the odds of advanced nodal stage (pN2b OR = 2.49; *p* = 0.008; pN3b OR = 2.65; *p* = 0.001). Contralateral lymphatic drainage occurred in 21.6% and metastases in 5.4% of patients.

**Conclusions:**

Preoperative [^18^F] FDG PET/CT nodal burden, tumor size and contralateral drainage patterns are potential predictors for patient-specific tailoring of the extent and laterality of neck dissection in TSCC.

**Clinical relevance:**

Quantifying nodal burden and integrating biological factors such as perineural invasion and sex-related differences may refine individualized surgical strategies and improve prognostic accuracy beyond conventional TNM staging.

## Introduction

Tongue carcinoma represents a clinically and prognostically distinct subset of oral squamous cell carcinoma (OSCC), characterized by a high rate of occult lymph node metastases, even in patients with a clinically negative neck (cN0) [1–5]. Clinical decision-making regarding type and extent of cervical lymph node dissection has significant impact on oncologic, prognostic and functional treatment outcomes [6, 7].

In TSCC the surgical management of contralateral cervical lymph node levels remains a subject of ongoing controversy, particularly in patients with a clinically negative neck (cN0) [8, 9]. TSCC drain into contralateral cervical lymph node in over 20% of patients [10, 11]. The frequency of this drainage pattern is primarily determined by the degree of tumor lateralization and its distance from the midline, in addition to the T stage [12, 13]. From a clinical perspective, delayed diagnosis of contralateral lymph node metastases is notably associated with a significantly reduced disease-specific five-year survival rate [14, 15]. This emphasizes the importance of refined risk stratification and individualized treatment planning.

In recent years, a paradigm shift has occurred toward patient-specific, morbidity-sparing treatment algorithms that integrate advanced imaging modalities with selective surgical strategies [16–18]. Patient-specific approaches increasingly rely on the preoperative use of high-sensitivity diagnostic tools such as fluorine-18 fluorodeoxyglucose ([^18^F] FDG) positron emission tomography combined with computed tomography (PET/CT) and lymphoscintigraphy [19–22]. When interpreted with clinical, pathological and staging information, these methods allow for a comprehensive assessment of lymphatic drainage and the identification of occult metastases, thereby facilitating a more precise and patient-specific risk stratification. Combined with minimally invasive procedures such as lymphoscintigraphy or sentinel lymph node biopsy (SLNB), these radioguided surgery strategies allow for targeted, less invasive surgical management while preserving oncologic safety [15, 23, 24].

For patients with early-stage tumors (cT1-T2, cN0), this diagnostic–therapeutic approach offers the opportunity to omit extensive elective neck dissections or to limit them to selected nodal levels [25]. The overarching goal is to reduce postoperative complications (e.g., shoulder dysfunction, sensory deficits and lymphedema), while ensuring precise locoregional tumor control [26–28].

The primary aim of this retrospective cohort study was to evaluate the surgical management of histologically confirmed TSCC with a particular focus on the extent of neck dissection. The secondary aim was to identify clinicopathological predictors of recurrence and survival to assess the role of imaging-guided selection and minimally invasive, function-preserving extent of neck dissection. The generated data may support the development of refined, patient-specific treatment pathways and informed decision-making in the era of personalized oncology.

## Materials and methods

### Study design and ethical approval

To address the study objectives, we conducted a retrospective cohort analysis. We reviewed the medical records of patients with histologically confirmed TSCC who underwent primary treatment at our Department of Oral and Plastic Maxillofacial Surgery between November 2011 and October 2024. Data were retrieved from the electronic medical records system, radiological information system (RIS) and German Center for Cancer Registry Data. Ethical approval for this investigation was obtained from the Ethics Committee of the Chamber of Physicians in Baden-Württemberg, University of Ulm, Germany (approval number: 162/22; date of approval: August 1, 2022). The study was designed in accordance with the Strengthening the Reporting of Observational Studies in Epidemiology (STROBE) guidelines and conducted in compliance with the principles of the Declaration of Helsinki (1964) and its subsequent amendments [29].

### Patient cohort

Patients were included if they met the following criteria: (I) histologically confirmed primary TSCC; (II) preoperative staging comprising a whole-body [^18^F] FDG PET/CT; (III)surgical management of TSCC, including tumor resection and unilateral or bilateral neck dissection; (IV) M0 status.

Exclusion criteria were: (I) recurrent TSCC; (II) non-surgical management or receipt of neoadjuvant therapy; (III) histopathological diagnoses other than TSCC or OSCC of other intraoral subsites; (IV) absence of [^18^F] FDG PET/CT in the preoperative staging; (V) incomplete medical records.

### Patient screening

Diagnostic codes from the ICD-10-GM (version 2024) classification system were used to identify patients diagnosed with TSCC (C01.9, C02.0, C02.1, C02.2, C02.3 and C02.9). Corresponding case numbers were used to retrieve electronic medical records from hospital’s digital clinical information system (Nexus^®^, Nexus AG, Donaueschingen, Germany), radiological information system (RIS) and image viewing software Visage Imaging^®^ (Visage Imaging Inc., San Diego, USA). Data collection was based on clinical, radiological and pathological reports from the Departments of Oral and Plastic Maxillofacial Surgery and Nuclear Medicine, Radiology, Institute for Pathology and German Center for Cancer Registry Data.

### Protocol of staging

All patients with suspected TSCC underwent a standardized inpatient staging protocol at our department. A board-certified oral and maxillofacial surgeon conducted a comprehensive clinical evaluation, including medical history, comprehensive examination of the oral cavity and cervical region and standardized photographic documentation. Definitive staging comprised whole-body [^18^F] FDG/CT and cervical ultrasonography. Ultrasonography was routinely scheduled for the day following [^18^F] FDG PET/CT to minimize cumulative nuclear exposure for hospital personnel and to streamline clinical workflow. All imaging studies were interpreted by board-certified radiologists and nuclear medicine specialists. Clinical and imaging findings were systematically integrated to determine primary tumor extent, cervical lymph node involvement and the presence of distant metastases.

Subsequently, surgical biopsy of the clinically and radiologically suspicious tongue lesion was performed under local anesthesia. Histopathological confirmation was established through microscopic and immunohistochemical analysis by a board-certified pathologist.

### Protocol of [^18^F] FDG PET/CT

Whole-body imaging was conducted after intravenous administration of 192 MBq [^18^F] fluorodeoxyglucose, with the dose individually adjusted according to body weight and blood glucose level. To improve image quality 10 mg of furosemide was administered intravenously prior to scanning. Acquisition commenced 80 min post-injection, using a Siemens Biograph Vision 600 (Definition Edge 128; Siemens Healthineers, Erlangen, Germany) system at 1.1 mm/sec for the torso. Iterative reconstruction was applied and quantitative analysis was performed using body-weight–adjusted Standardized Uptake Value maximum (SUV_max_) with a 50% isocontour Volume-of-interest (VOI) method (cm³). Response assessment followed Response Evaluation Criteria in Solid Tumors (RECIST) 1.0 criteria [30]. CT was performed with oral and intravenous contrast (122 ml, 300 mg iodine/ml; venous phase). The scan range extended from the skull vertex to the proximal femur. Data analysis included computer-assisted 3D evaluation with segmental multiplanar reconstruction (3 mm slice thickness; lung, soft-tissue and bone windows) in mid-respiratory position plus a low-dose inspiratory lung CT for quantification. Reference SUV_max_ values were the liver and aortic blood pool. [^18^F] FDG PET/CT was performed according to a standardized institutional protocol. Images were reviewed by board-certified radiologists and nuclear medicine specialists. Evaluation included the brain, head and neck, thorax, abdomen, pelvis and skeleton. In consideration of these findings, a clinical (c)TNM classification of the primary TSCC was formulated, derived primarily from radiological assessments.

Preoperative [¹⁸F] FDG PET/CT scans were evaluated using combined visual and semiquantitative criteria, following the methodology described in our previous work [31]. Focal FDG uptake was considered suspicious when accompanied by morphological abnormalities (e.g., heterogeneous density, irregular borders or rim enhancement) regardless of nodal size. SUV_max_ values were used to support visual assessment but not as a strict diagnostic threshold, given interindividual and interscanner variability. For each positive neck level, number and location of involved nodes were recorded. If multiple lymph nodes were present, the highest SUV_max_ within that level was documented. Due to inconsistencies in SUV acquisition across the study period (2011–2024), SUV_max_ and metabolic nodal volumes were not included as predictor variables in the statistical modelling.

### [^18^F] FDG PET/CT assessment of suspicious lymph nodes

Preoperative [¹⁸F] FDG PET/CT scans were evaluated using a combined assessment, following the criteria established in our previously validated institutional [^18^F] FDG PET/CT workflow [31]. To ensure methodological consistency, the same definitions were applied in the present study.

Lymph nodes were classified as suspicious based on the following criteria. (I) Semiquantitative threshold: Lymph nodes were deemed [^18^F] FDG PET/CT-positive, if the measured uptake exceeded the institutional cutoff of SUV > 5, which also served as the threshold for classifying primary tumors as metabolically active. (II) Morphological criteria: Nodes with SUV values ≤ 5 were considered suspicious, if morphological features suggestive of metastasis were present, including central necrosis, round configuration with a short-to-long axis ratio approaching 1.0, enlargement or loss of the fatty hilum according to RECIST criteria [32]. (III) Classification framework: Clinical nodal staging (cN) was assigned according to the guideline classification for squamous cell carcinoma of the oral cavity applicable during the study period [33]. Suspicious lymph nodes identified by these criteria were used to quantify preoperative nodal burden and to assess laterality of lymphatic drainage.

### Multidisciplinary head and neck tumor board

After completion of diagnostic staging, all cases were presented in the interdisciplinary head and neck tumor board of our certified cancer center. The board reviewed all available clinical, radiological and histopathological findings to establish a patient-specific treatment plan. Particular attention was given to the extent of neck dissection, reconstructive strategies and indications for adjuvant therapy. Recommendations were based not only on tumor characteristics and disease stage but also on patient-specific factors, including comorbidities, functional status and individual treatment preferences.

### Surgical procedure

Supraomohyoid neck dissection (SOHND) was performed according to oncologic guidelines, encompassing levels I-III while preserving the spinal accessory nerve, internal jugular vein and sternocleidomastoid muscle with extension to level IV as indicated by clinical or intraoperative findings [34–37]. In cN0 TSCC, SOHND of levels I–III was the standard approach. All procedures were performed under general anesthesia in the same session as primary tumor resection and reconstruction, with neck dissection carried out immediately following tumor removal.

### Definition of recurrence categories and salvage management

Recurrence was categorized into predefined groups [14, 38, 39]: (I) local recurrence, defined as tumor reappearance at or adjacent to the primary site following initial tumor resection; (II) regional recurrence, defined as metastatic reappearance within the ipsilateral cervical lymph nodes following initial neck dissection; (III) contralateral lymph node recurrence, defined as new metastatic involvement of the contralateral neck after primary treatment; (IV) distant recurrence defined as metastatic spread to non-regional sites abroad the head and neck regio (e.g., lung, liver, bone). All four categories taken together were defined as total recurrence (V). These definitions were applied consistently throughout the analysis.

Local and regional recurrences were evaluated in a multidisciplinary tumor board and treated with salvage surgery whenever feasible. Decisions regarding adjuvant radiotherapy or chemoradiotherapy were based on established pathological risk factors [33]. In cases of contralateral nodal recurrence, contralateral neck dissection was performed, followed by adjuvant therapy when indicated [33].

### Data collection

All patients were anonymized before data analysis. Data were obtained from hospital records and included demographics (e.g., age, sex), risk factors (e.g., tobacco and alcohol consumption), preoperative tumor staging findings (ultrasonography and [^18^F] FDG PET/CT, including SUV_max_, nodal involvement and presence of distant metastases), tumor characteristics (e.g., primary site, postoperative histopathological TNM stage, histopathological grade, perineural invasion, lymphovascular invasion and margin status) and surgical details (e.g., type, extent and laterality of neck dissection, intraoperative findings). Histopathological results recorded the number of lymph nodes resected, location and extent of metastases, extracapsular extension (ECE), micrometastases and isolated tumor cells. Depth of invasion (DOI) was not systematically documented in pathology reports throughout the study period and could therefore not be included as a continuous variable. Because DOI is already integrated in the AJCC 8th edition pT classification, pT stage used in all analyses indirectly reflects DOI-based tumor invasiveness [40]. As the primary aim of this study was to evaluate for neck dissection decision-making, DOI (as a postoperative parameter) was not part of the predictor set.

### Study variables

The predictor and outcome variables are presented in Table 1.

**Table 1:** Predictor variables and analyzed clinical outcome variables in TSCC

Predictor Variables	Outcome Variables
Pathological T stage (pT)	• Ipsilateral lymph node metastasis • Tumor localization • Local (I) and regional (II) recurrence
Pathological N stage (pN) and number of ipsilateral metastatic lymph nodes	• Risk of contralateral lymph node recurrence (III)
Extent of neck dissection (ipsilateral vs. bilateral) in combination with pN stage	• OS • Risk of contralateral lymph node recurrence (III) in advanced TSCC (≥ pT3)
Tumor size (mm) and pN stage	• OS
Clinically suspicious cervical lymph nodes (cN) on [^18^F] FDG PET/CT	• Association with pN stage
Total number of metastatic lymph nodes (pN+)	• OS • Local (I) and r egional (II) recurrence
pT stage and pN stage	• Local (I) and regional (II) recurrence
Regional recurrence in pN0 patients	• Relation to pT stage

Overview of all predictor variables included in the statistical analyses and their corresponding outcome variables. Potential confounding variables considered across relevant analyses included date of surgery, tumor size (mm), N stage, lymphatic drainage characteristics and the number of suspicious cervical lymph nodes.

Abbreviations: *OS* overall survival, *pT* pathological tumor stage, *pN* pathological nodal stage, *cN* clinically suspicious cervical lymph nodes, *[18 F] FDG PET/CT* fluorine-18 fluorodeoxyglucose positron emission tomography/computed tomography.

### Statistical analysis

Data were centralized in an electronic format using Microsoft Excel (Microsoft Corporation, Redmond, USA) and analyzed using R software (R Foundation for Statistical Computing, Vienna, Austria). Descriptive statistics were applied to summarize baseline characteristics. Categorical variables are presented as absolute and relative frequencies, while continuous variables are reported as means with standard deviations.

Normality of distribution was assessed with the Shapiro-Wilk test, evaluation of kurtosis, skewness and graphical inspection. Predictor variables included in the regression models were selected a priori based on clinical relevance, biological plausibility and previously published evidence on factors influencing cervical nodal metastasis of TSCC. Depending on distribution, group comparisons were performed using Student’s t-test or Mann-Whitney U test and paired data were analyzed with paired t-test or Wilcoxon signed-rank test. Fisher’s exact test was applied for contingency tables with small sample sizes. Correlations were examined with Pearson’s or Spearman’s coefficients, as appropriate and effect sizes were classified into weak, moderate, high and very high. Analysis of variance (ANOVA) was performed using the R function aov() and multiple comparisons were adjusted with Holm’s correction. Regression models were used for multivariable analyses, with odds ratios reported where applicable. Logistic regression was used to analyze the relationship between predictor variables and deceased (Table 1). It was found that, holding all other predictor variables constant, the odds of decease occurring increased by some percent] (95% CI [Lower Limit, Upper Limit]) for a one-millimeter increase in tumor diameter.

Diagnostic accuracy metrics of [^18^F] FDG PET/CT [e.g., sensitivity, specificity, positive predictive value (PPV) and negative predictive value (NPV)] were not calculated for the present study, as these parameters have already been comprehensively validated for the identical institutional [^18^F] FDG PET/CT workflow [31]. Given the very limited number of outcome events in our cohort (e.g., decease and contralateral metastases; Table 2), model complexity was deliberately restricted to prevent overfitting. Accordingly, only a limited number of predictors was included per model. In the presence of complete or quasi-complete separation and to obtain bias-reduced and numerically stable effect estimates, Firth’s penalized maximum-likelihood logistic regression was applied [41]. Because of the minor event counts and inherent model constraints, all regression results should be interpreted as exploratory rather than confirmatory.

**Table 2:** Baseline characteristics of the study cohort (*n* = 74)

Characteristics	*n*	%	Mean (SD)	Median (range)
Age	—	—	61.7 (13.4)	60.72 (28.9–88.3)
Sex				
Male	40	54.05	—	—
Female	34	45.95	—	—
Tumor site				
Tongue base	1	1.35	—	—
Lateral tongue	66	89.19	—	—
Tongue + anterior floor	7	9.46	—	—
Tumor side				
Left	42	56.76	—	—
Right	32	43.24	—	—
Localization				
Anterior	19	25.68	—	—
Anterior + posterior	27	36.49	—	—
Posterior	28	37.84	—	—
cT stage				
cTx	11	14.86	—	—
cT0	2	2.7	—	—
cT1	26	35.14	—	—
cT2	28	37.84	—	—
cT3	6	8.11	—	—
cT4a	1	1.35	—	—
cN stage				
cNx	0	0.0		
cN0	39	52.7	—	—
cN1	12	16.22	—	—
cN2a	1	1.35	—	—
cN2b	17	22.97	—	—
cN2c	5	6.76	—	—
pT stage				
pTx	1	1.35	—	—
pTis	2	2.7	—	—
pT1	32	43.24	—	—
pT2	29	39.2	—	—
pT3	10	13.51	—	—
pN stage				
pNx	4	5.4	—	—
pN0	41	55.41	—	—
pN1	6	8.11	—	—
pN2a	1	1.35	—	—
pN2b	7	9.46	—	—
pN2c	3	4.05	—	—
pN3b	12	16.22	—	—
Recurrence (I - V)				
Yes	20	27.03	—	—
No	54	72.97	—	—
Recurrence-free survival	—	—	37 (39.1)	19 (2–154)
Survival				
Positive	65	87.84	—	—
Deceased	9	12.16	—	—
Survival time	—	—	48.1 (45.4)	26.5 (2–157)

Patient demographics, tumor site, localization, clinical and pathological staging are summarized. Recurrence [local (I), regional (II), contralateral nodal (III), distant (IV) and total recurrence (V)], recurrence-free survival (RFS), survival and survival time are reported. Values are given as absolute numbers; continuous variables are presented as mean ± standard deviation (SD) with median and range. Age is reported in years and survival time in months.

Abbreviations: *cT* clinical tumor stage, *cN* clinical nodal stage, *pT* pathological tumor stage, *pN* pathological nodal stage, *SD* standard deviation; definition of recurrence categories: (I) local recurrence, defined as tumor reappearance at or adjacent to the primary site following initial tumor resection; (II) regional recurrence, defined as metastatic reappearance within the ipsilateral cervical lymph nodes following initial neck dissection; (III) contralateral nodal recurrence, defined as new metastatic involvement of the contralateral neck after primary treatment; *RFS* recurrence-free survival, *OS* overall survival.

## Results

### Baseline characteristics

A total of 74 patients were included in this retrospective analysis, with a mean age at initial staging of 61.7 ± 13.4 years (range, 28.9–88.3). The cohort consisted of 34 females (45.95%) and 40 males (54.05%). Most tumors (*n* = 66; 89.19%) were located at the lateral border of the tongue. 7 cases (9.46%) involved the tongue with extension into the anterior floor of the mouth (FOM). One tumor (1.35%) was located at the tongue base. Baseline characteristics of the study cohort are summarized in Table 2.

### Predictors of ipsi- and contralateral lymphatic drainage

Of the 74 patients, 41 (55.4%) presented without cervical metastases, whereas 33 (44.6%) had histologically confirmed lymph node involvement (Table 3). Ipsilateral metastases occurred in 22 patients, while additional contralateral lymphatic drainage was observed in 11 cases (21.6%). Contralateral involvement was not significantly associated with sex (male 7/25; 28% vs. female 4/20; 20%; *p* = 0.73) or tumor site, occurring mainly in lateral tongue carcinomas (10/42; 23.8%) and less frequently in tumors with anterior floor extension (1/2; 50%), but not in tongue base carcinomas (0/1; 0%; *p* = 0.58).

**Table 3:** Clinicopathological factors associated with ipsi- and contralateral lymphatic drainage

Variables	Ipsilateral *n*/*N* (%)	Contralateral + ipsilateral *n*/*N* (%)	*p*-value
Sex			0.73
Male	18/25 (72)	7/25 (28)	
Female	16/20 (80)	4/20 (20)	
Tumor site			0.58
Tongue base	1/1 (100)	0/1 (0)	
Lateral tongue + anterior floor	1/2 (50)	1/2 (50)	
Lateral tongue	32/42 (76.2)	10/42 (23.8)	
Ventral tongue + anterior floor	0/0 (0)	0/0 (0)	
Grade			1.0
G1	0/0 (0)	0/0 (0)	
G2	19/26 (73.1)	7/26 (26.9)	
G3	13/17 (76.5)	4/17 (23.5)	
G4	2/2 (100)	0/2 (0)	
pT stage			0.16
pTx	0/1 (0)	1/1 (100)	
pTis	0/0 (0)	0/0 (0)	
pT1	13/19 (68.4)	6/19 (31.6)	
pT2	16/20 (80.0)	4/20 (20.0)	
pT3	5/5 (100)	0/5 (0)	
pN stage			0.4
pNx	0/0 (0)	0/0 (0)	
pN0	20/28 (71.4)	8/28 (28.6)	
pN1	2/3 (66.7)	1/3 (33.3)	
pN2a	1/1 (100)	0/1 (0)	
pN2b	4/4 (100)	0/4 (0)	
pN2c	0/1 (0)	1/1 (100)	
pN3b	7/8 (87.5)	1/8 (12.5)	
PNI			0.4
Absent	23/32 (71.9)	9/32 (28.1)	
Present	9/10 (90)	1/10 (10)	
L			0.45
Absent	20/28 (71.4)	8/28 (28.6)	
Present	12/14 (85.7)	2/14 (14.3)	

Data are presented as absolute numbers with percentages in parentheses. p-values were calculated using Fisher’s exact test. Statistical significance was set at *p* ≤ 0.05. Levels of significance are indicated as follows: * *p* ≤ 0.05; ** *p* ≤ 0.01; *** *p* ≤ 0.001.

Abbreviations: *n/N* number of positive cases within subgroup/total cases, *pT* pathological tumor stage, *pN* pathological nodal stage, *PNI* perineural invasion, *L* lymphovascular invasion.

### Predictors of recurrence

**Fig. 1:**
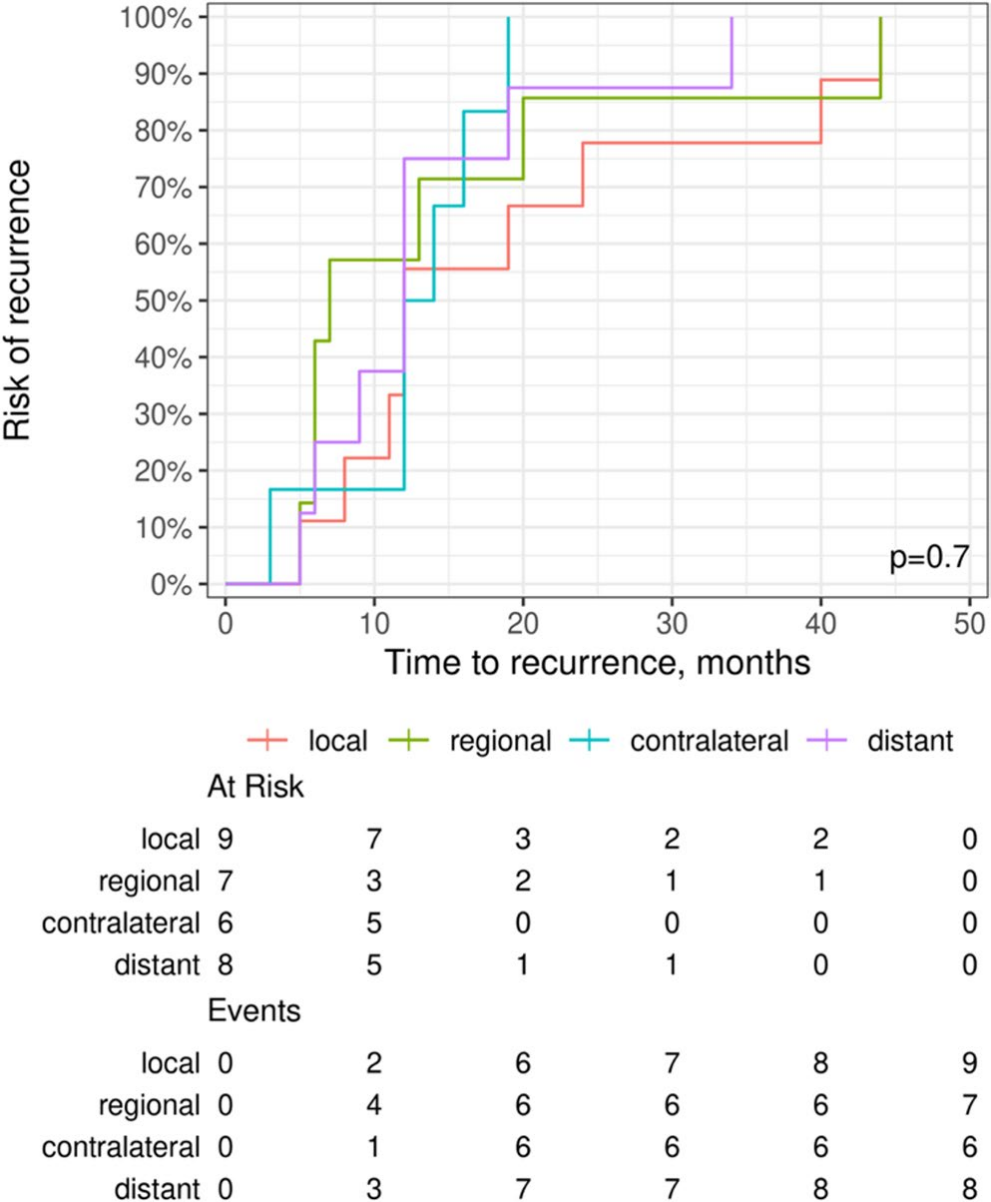
Time to recurrence stratified by recurrence pattern (median OS: 26.5 months; median RFS: 19.0 months). Kaplan-Meier curves illustrate recurrence-free survival according to the anatomical site of first recurrence [local (I), regional (II), contralateral (III) and distant (IV) recurrence]. No statistically significant difference in time-to-recurrence distributions was observed between groups (*p* = 0.7). Numbers at risk and cumulative events at predefined time points are shown below the plot

**Fig. 2:**
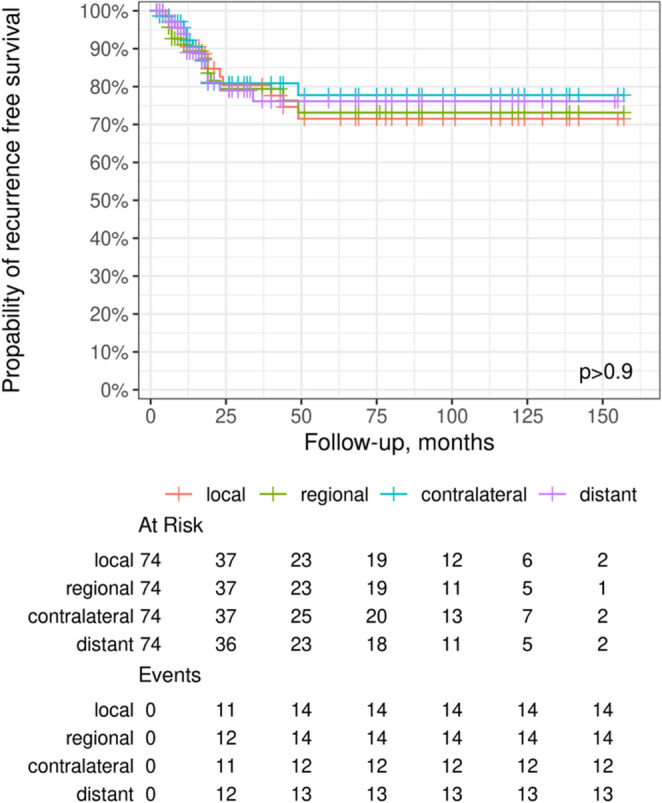
Kaplan-Meier curves for recurrence-free survival (RFS) stratified by recurrence pattern (median OS: 26.5 months; median RFS: 19.0 months). RFS probabilities are shown for patients with local (I), regional (II), contralateral nodal (III) and distant (IV) recurrence. No significant difference in RFS was observed among the four recurrence patterns (*p* > 0.9). Numbers at risk and event counts for each recurrence category are displayed below the plot

Total recurrence (V) was observed in 20 of 74 patients (27%). Univariate analysis showed a trend toward higher recurrence rates in females (*p* = 0.066), advanced pN stage (*p* = 0.1) and in cases with perineural invasion (*p* = 0.095), without reaching statistical significance (Table 4). In Kaplan-Meier analyses, time to recurrence did not differ significantly between recurrence patterns (local, regional, contralateral and distant; log-rank test, *p* = 0.7; Fig. 1). The anatomical site of recurrence was not significantly associated with recurrence-free survival (log-rank test, *p* > 0.9; Fig. 2).

**Table 4:** Analysis of clinicopathological predictors associated with local tumor recurrence in TSCC patients

Variables	Recurrence *n*/*N* (%)	No recurrence *n*/*N* (%)	*p*-value
Sex			
Male	7/40 (17.5)	33/40 (82.5)	0.066
Female	13/34 (38.2)	21/34 (61.8)	
Tumor site			
Tongue base	0/1 (0)	1/1 (100)	0.35
Lateral tongue	17/66 (25.8)	49/66 (74.2)	
Lateral tongue + anterior floor	0/2 (0)	2/2 (100)	
Ventral tongue + anterior floor	3/5 (60)	2/5 (40)	
Grade			
G1	1/2 (50)	1/2 (50)	0.15
G2	7/41 (17.1)	34/41 (82.9)	
G3	10/27 (37)	17/27 (63)	
G4	1/3 (33.3)	2/3 (66.7)	
pT stage			
pT1	8/32 (25)	24/32 (75)	0.19
pT2	6/29 (20.7)	23/29 (79.3)	
pT3	6/10 (60)	4/10 (40)	
pTis/pTsin1/pTx	0/3 (0)	3/3 (100)	
pN stage			
pN0	7/41 (17.1)	34/41 (82.9)	0.1
pN1	1/6 (16.7)	5/6 (83.3)	
pN2a	0/1 (0)	1/1 (100)	
pN2b	3/7 (42.9)	4/7 (57.1)	
pN2c	2/3 (66.7)	1/3 (33.3)	
pN3b	6/12 (50)	6/12 (50)	
Not reported	1/4 (25)	3/4 (75)	
PNI			
Absent	10/52 (19.2)	42/52 (80.8)	0.095
Present	7/16 (43.8)	9/16 (56.2)	
L			
Absent	9/43 (20.9)	34/43 (79.1)	0.39
Present	8/25 (32)	17/25 (68)	
Neck dissection			
Bilateral	18/65 (27.7)	47/65 (72.3)	0.92
Unilateral/none/SLNB	2/7 (28.6)	5/7 (71.4)	

Univariate analysis of clinicopathological predictors of local tumor recurrence in patients with TSCC. Recurrence rates are presented by sex, tumor site, histological grade, pathological T and N stage, perineural and lymphovascular invasion and extent of neck dissection. p-values are derived from Fisher’s exact tests. Statistical significance was set at *p* < 0.05. Levels of significance are indicated as follows: * *p* ≤ 0.05; ** *p* ≤ 0.01; *** *p* ≤ 0.001.

Abbreviations: *n/N* number of positive cases within subgroup/total cases, *pT* pathological tumor stage, *pN* pathological nodal stage, *PNI* perineural invasion, *L* lymphovascular invasion, *SLNB* sentinel lymph node biopsy

### Predictors of overall survival

At the end of median follow-up (26.0 months), 65 of 74 patients (87.8%) survived and 9 (12.2%) did not survive (Table 5). Overall survival (OS) did not differ significantly by sex. There was a trend toward reduced OS for tumors of the ventral tongue with anterior floor involvement and for higher tumor grades. A similar trend was observed for increasing pN stages. Importantly, the presence of PNI was significantly associated with worse OS (PNI_Absent_: 92.3% vs. PNI_Present_: 68.8%; p = 0.03). Other variables, including tumor site, pathological T stage and lymphovascular invasion, were not significantly related to OS.

**Table 5:** Association of clinicopathological variables with overall survival in patients with TSCC

Variables	Survivors *n*/*N* (%)	Non survivors *n*/*N* (%)	*p*-value
Sex			0.72
Male	36/40 (90)	4/40 (10)	
Female	29/34 (85.3)	5/364(14.7)	
Tumor site			0.073
Tongue base	1/1 (100)	0/1 (0)	
Lateral tongue + anterior floor	1/2 (50)	1/2 (50)	
Lateral tongue	60/66 (90.9)	6/66 (9.1)	
Ventral tongue + anterior floor	3/5 (60)	2/5 (40)	
Grade			0.072
G1	2/2 (100)	0/2 (0)	
G2	39/41 (95.1)	2/41 (4.9)	
G3	20/27 (74.1)	7/27 (25.9)	
G4	3/3 (100)	0/3 (0)	
pT stage			0.42
pTx	1/1 (100)	0/1 (0)	
pTis	2/2 (100)	0/2 (0)	
pT1	30/32 (93.8)	2/32 (6.3)	
pT2	25/29 (86.2)	4/29 (13.8)	
pT3	7/10 (70)	3/10 (30)	
pN stage			0.077
pNx	4/4 (100)	0/4 (0)	
pN0	39/41 (95.1)	2/41 (4.9)	
pN1	5/6 (83.3)	1/6 (16.7)	
pN2a	1/1 (100)	0/1 (0)	
pN2b	5/7 (71.4)	2/7 (28.6)	
pN2c	3/3 (100)	0/3 (0)	
pN3b	8/12 (66.7)	4/12 (33.3)	
PNI			0.03*
Absent	48/52 (92.3)	4/52 (7.7)	
Present	11/16 (68.8)	5/16 (31.2)	
L			0.26
Absent	39/43 (90.7)	4/43 (98.9)	
Present	20/25 (80)	5/25 (20)	

Overall survival (OS) rates are presented by sex, tumor site, histological grade, pathological T and N stage, perineural and lymphovascular invasion. p-values were calculated using Fisher’s exact test. Statistical significance was set at *p* ≤ 0.05. Levels of significance are indicated as follows: * *p* ≤ 0.05; ** *p* ≤ 0.01; *** *p* ≤ 0.001.

Abbreviations: *n/N* number of positive cases within subgroup/total cases, *pT* pathological tumor stage, *pN* pathological nodal stage, *PNI* perineural invasion, *L* lymphovascular invasion.

### Survival analysis

The 1-year and 5-year survival rates for the entire cohort (*n* = 74) were 97% (95% CI: 0.93–1.00) and 83% (95% CI 0.73–0.94), respectively. At the last median follow-up (26.0 months), 65 patients (87.8%) survived and 9 (12.2%) did not survive. There was a trend toward worse survival in patients with contralateral metastases (5-year survival: 67% vs. 84%). Similarly, higher pN stage was associated with reduced 5-year survival, without significance (*p* = 0.077). Perineural invasion was significantly associated with poorer survival (5-year survival: 65% vs. 88%; *p* = 0.03), whereas extracapsular extension (ECE) and lymphovascular invasion did not markedly affect 5-year survival.

### Recurrence-free survival

**Fig. 3:**
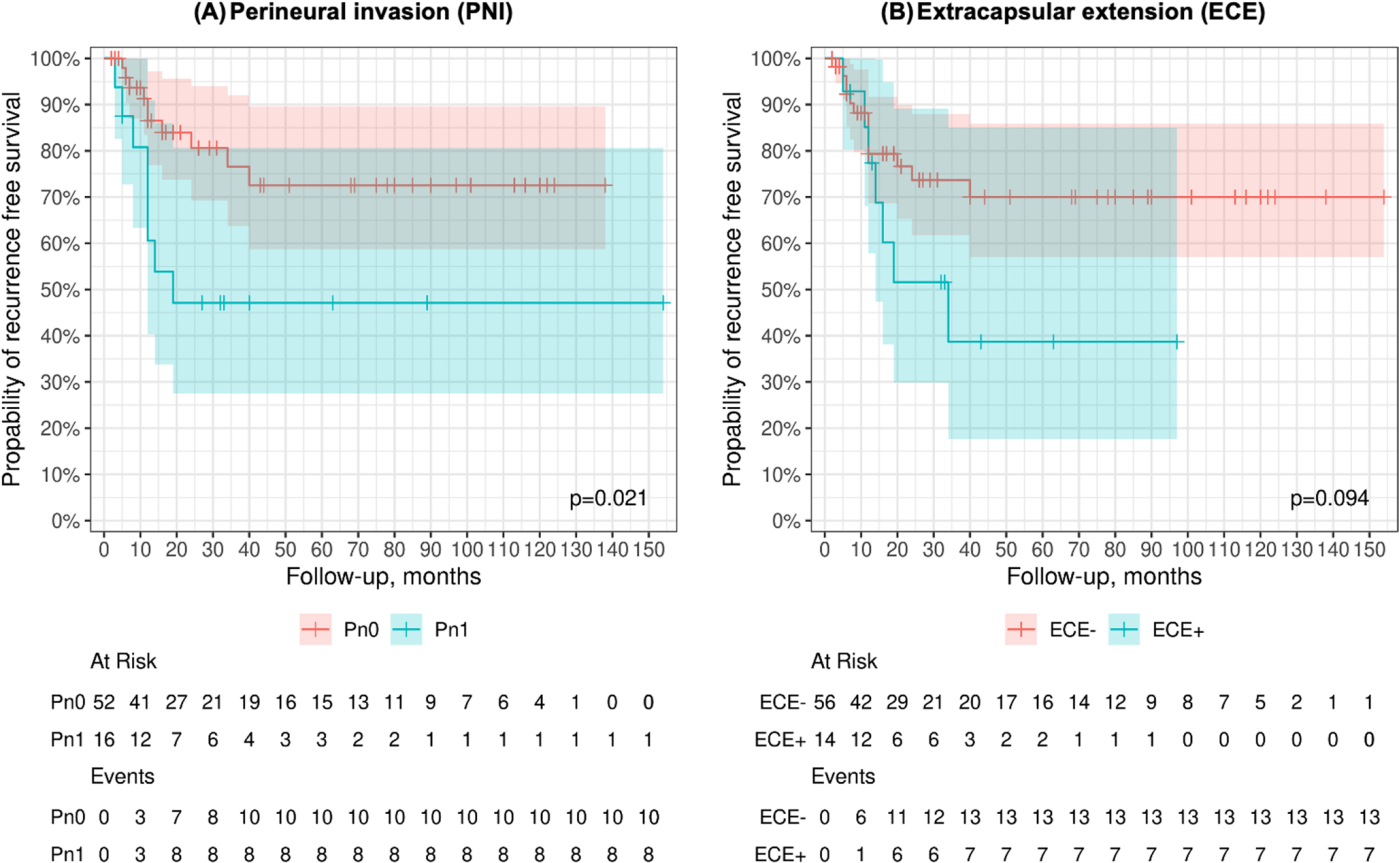
Impact of biological tumor characteristics on recurrence-free survival (RFS) in patients with TSCC (median OS: 26.5 months; median RFS: 19.0 months). Impact biological features on recurrence-free survival (RFS) in patients with TSCC [median overall survival (months): 26.5; median recurrence-free survival (months): 19.0]. Kaplan-Meier survival curves illustrating 12-month and 5-year RFS rates stratified by (**A**) perineural invasion (PNI) and (**B**) extracapsular extension (ECE). Patients without PNI (group 0) showed significantly improved 5-year RFS compared to those with PNI (group 0 = 73% vs. group 1 = 47%, *p* = 0.021). In contrast, the presence of ECE (group 1) was associated with a markedly reduced 5-year survival (group 1 = 39% vs. group 0 = 70%), although this difference did not reach statistical significance (*p* = 0.094). Shaded areas represent 95% CI. Statistical significance was set at p **≤** 0.05. Levels of significance are indicated as follows: * *p* ≤ 0.05; ** *p* ≤ 0.01; *** *p* ≤ 0.001. Abbreviations: PNI = perineural invasion; ECE = extracapsular extension; RFS = recurrence-free survival; CI = confidence interval; p = p-value

The 1-year and 5-year RFS rates were 80% (95% CI: 0.70–0.90) and 61% (95% CI: 0.49–0.77), respectively. Females tended to less favorable RFS (5-year RFS: 47% vs. 74% in men). Contralateral nodal disease and perineural invasion were associated with reduced RFS (5-year RFS: 50% and 47%, respectively). The presence of extracapsular extension also correlated with worse RFS (5-year RFS: 39% vs. 70% in ECE-; Fig. 3).

### Association between pT stage and ipsilateral lymph node metastasis

A highly significant association was identified between pT stage and ipsilateral lymph node involvement (*p* = 0.0003), indicating that the likelihood of nodal metastasis increases systematically with advancing tumor stage.

### Association between pT stage and tumor localization

pT stage was significantly associated with anatomical subsite, with a markedly different distribution of stages across subsites (*p* < 0.0001). Tumors located in the anterior region were predominantly early stage (pT1–2), whereas lesions extending across anterior and posterior regions were mainly advanced (pT2–3). Posterior tumors exhibited a heterogeneous pattern, most frequently classified pT1–2.

### Association between ipsilateral node count and contralateral lymph node metastasis

A trend was observed toward higher rates of contralateral metastasis in patients with an increasing number of ipsilateral metastatic nodes. However, the limited number of contralateral cases (*n* = 4) did not allow this finding to reach statistical significance (*p* = 0.072).

### Descriptive analysis of mortality in TSCC

Mortality was descriptively analyzed using Fisher’s exact test to account for the limited number of outcome events (Table 6). No statistically significant association was observed between the type or laterality of neck dissection and mortality (*p* = 1.0). All mortality events occurred in patients undergoing bilateral neck dissection, whereas no deaths were observed among patients without neck dissection, those treated with unilateral neck dissection, or those undergoing sentinel lymph node biopsy (SLNB). With regard to the tumor stage, no statistically significant association between pT stage and mortality was detected (*p* = 0.42). Although numerically higher numbers of deaths were observed in patients with more advanced pT stages, mortality events occurred across multiple tumor stages, precluding reliable inferential conclusions. In contrast, nodal status demonstrated a borderline association with mortality (*p* = 0.077). Deaths were more frequently observed in patients with advanced nodal disease. This pattern suggests a clinically relevant trend toward increased mortality with increasing nodal burden; however, due to the limited number of events and sparse distribution across nodal subcategories, this observation must be interpreted with caution.

**Table 6:** Fisher’s exact test for descriptive analysis of mortality in patients with TSCC

Variables	Survived	Deceased	*p*-value
Neck dissection			1.0
None	4	0	
Bilateral	56	9	
Right	2^†^	0	
Left	2	0	
SLNB right	1	0	
SLNB left	1^†^	0	
pTstage			0.42
pTx	1	0	
pTis	2	0	
pT1	30	2	
pT2	25	4	
pT3	7	3	
pN stage			0.077
pNx	4	0	
pN0	39	2	
pN1	5	1	
pN2a	1	0	
pN2b	5	2	
pN2c	3	0	
pN3b	8	4	

Descriptive analysis of mortality in patients with tongue squamous cell carcinoma (TSCC) using Fisher’s exact test. Mortality is shown according to neck dissection status, primary tumor stage (pT) and nodal stage (pN). Due to the limited number of events, results are presented descriptively. Statistical significance was set at p ≤ 0.05. Levels of significance are indicated as follows: * *p* ≤ 0.05; ** *p* ≤ 0.01; *** *p* ≤ 0.001.

For the descriptive analysis of the category “Neck dissection,” a total of 75 procedures (*n* = 74) were considered, as one patient underwent ipsilateral neck dissection and contralateral sentinel lymph node biopsy due to bilateral lymphatic drainage; this case is marked with †.

Abbreviations: *pT* pathological tumor stage, *pN* pathological nodal stage, *SLNB* sentinel lymph node biopsy.

### Prognostic influence of total number of metastatic lymph nodes on survival

Binary logistic regression analysis was performed to evaluate the impact of the total number of metastatic lymph nodes (pN+) and the interval between diagnosis and surgery on overall survival (Table 7). Decease was defined as the dependent variable. The analysis demonstrated that the total number of pN + was a significant predictor of mortality (OR = 1.5; 95% CI: 1.08–2.16; *p* = 0.018), indicating that each additional metastatic lymph node increased the odds of decease by approximately 50%. In contrast, the time interval between diagnosis and surgery showed no significant effect on survival (OR = 0.99; 95% CI: 0.94–1.02; *p* = 0.64).

**Table 7:** Regression analysis of potential predictors of mortality in patients with TSCC

Variables	OR	95% CI	*p*-value
Total number of pN+	1.5	1.08–2.16	0.018*
Interval diagnosis to surgery (days)	0.99	0.94–1.02	0.64

Statistical significance was set at *p* < 0.05. Levels of significance are indicated as follows: * *p* ≤ 0.05; ** *p* ≤ 0.01; *** *p* ≤ 0.001.

Abbreviations: *OR* odds ratio, *CI* confidence interval, *pN +* pathologically positive lymph nodes.

### Descriptive analysis of recurrence in TSCC

Tumor recurrence was descriptively analyzed using Fisher’s exact test to account for the limited number of recurrence events (Table 8). No statistically significant association was observed between recurrence and the type or laterality of neck dissection (*p* = 0.92). Recurrence events occurred predominantly in patients undergoing bilateral neck dissection, whereas only isolated recurrence events were observed in patients without neck dissection. No recurrences were observed following sentinel lymph node biopsy (SLNB) procedures or unilateral neck dissection. Given the low number of events and the sparse distribution across categories, these findings should be interpreted descriptively.

**Table 8:** Fisher’s exact test for descriptive analysis of recurrence in patients with TSCC

Variables	Recurrence	No recurrence	*p*-value
Neck dissection			0.92
None	1	3	
Bilateral	18	47	
Right	0	2^†^	
Left	1	1	
SLNB right	0	1	
SLNB left	0	1^†^	
pTstage			0.19
pTx	0	1	
pTis	0	2	
pT1	8	24	
pT2	6	23	
pT3	6	4	
pN stage			0.1
pTx	1	3	
pN0	7	34	
pN1	1	5	
pN2a	0	1	
pN2b	3	4	
pN2c	2	1	
pN3b	6	6	

Descriptive analysis of recurrence in patients with tongue squamous cell carcinoma (TSCC) using Fisher’s exact test. Recurrence status is shown according to neck dissection strategy, primary tumor stage (pT) and nodal stage (pN). Owing to the limited number of recurrence events, results are presented descriptively. For the descriptive analysis of the category “Neck dissection,” a total of 75 procedures (n = 74) were considered, as one patient underwent ipsilateral neck dissection and contralateral sentinel lymph node biopsy due to bilateral lymphatic drainage; this case is marked with †.

Statistical significance was set at *p* ≤ 0.05. Levels of significance are indicated as follows: * *p* ≤ 0.05; ** *p* ≤ 0.01; *** *p* ≤ 0.001.

Abbreviations: *pT* pathological tumor stage, *pN* pathological nodal stage, *SLNB* sentinel lymph node biopsy.

With respect to the primary tumor stage, Fisher’s exact test did not reveal a statistically significant association between pT stage and recurrence (*p* = 0.19). Although numerically higher recurrence rates were observed in patients with advanced pT stages, recurrence events occurred across multiple tumor stages. Regarding nodal status, no statistically significant association between pN stage and recurrence was detected (*p* = 0.1). Recurrence events were observed in both node-negative and node-positive disease, including advanced nodal stages.

## Discussion

In this retrospective cohort study, we evaluated clinicopathological predictors of lymphatic dissemination, recurrence and survival to refine cervical management and support patient-specific treatment strategies of patients with tongue squamous cell carcinoma (TSCC).

TSCC represents the most common malignancy of the oral cavity and is characterized by early and aggressive lymphatic dissemination an occult nodal metastasis, even in early stages [1, 42]. Regional lymph node metastasis represents the pivotal prognostic predictor, exerting a profound impact on both recurrence risk and overall survival (OS) [43]. Accordingly, optimal cervical management has been a subject of enduring debate, particularly in cN0 necks, where the balance between oncologic safety and functional preservation is critical [2].

Over recent decades, advances in diagnostic imaging, sentinel lymph node biopsy (SLNB) and refined neck dissection techniques have improved staging accuracy and enabled more selective cervical management. Nevertheless, debate continues regarding the optimal extent and laterality of neck dissection, the prognostic value of nodal burden and the integration of biological risk factors and advanced imaging into therapeutic algorithms [44].

The diagnostic accuracy of our institutional [^18^F] FDG PET/CT protocol for preoperative staging, including sensitivity and specificity for the detection of metastatic cervical lymph nodes, has already been established in our previous validation study [31]. In this analysis [^18^F] FDG PET/CT imaging demonstrated a sensitivity of 59%, specificity of 87% and diagnostic accuracy of 77%. Cervical ultrasonography, in contrast, showed superior sensitivity (76%) but minor specificity (67%), while combined examination with [^18^F] FDG PET/CT and ultrasonography assessment improved sensitivity (78%) and negative predictive value (82%). Furthermore, although metastatic nodes exhibited higher SUV_max_ and RECIST short-axis values on average, neither metric provided a reliable diagnostic cut-off due to substantial overlap between benign and malignant nodes. Given that these diagnostic accuracy metrics have already been comprehensively validated for our institutional [^18^F] FDG PET/CT workflow, the present study builds on this established diagnostic foundation by shifting the focus toward prognostic implications of [^18^F] FDG PET/CT-derived nodal burden, specifically its value in predicting survival outcomes and for guiding preoperative decisions regarding the extent and laterality of neck dissection for tumors located close to the facial midline (e.g., TSCC).

In our cohort, the extent of clinical nodal burden was closely linked to prognostic outcomes and represented one of the most informative preoperative risk markers. The number of suspicious nodes detected on [^18^F] FDG PET/CT was the strongest predictor of advanced pathological nodal stages (pN2b and pN3b), underscoring the critical role of precise clinical and radiological assessment in surgical planning [45]. According to current guidelines, elective neck dissection (END) is recommended in cN0 TSCC when histopathological risk factors are present. In experienced centers, SLNB may be considered as an alternative approach [37, 46]. In this context, robust preoperative clinicopathological and imaging predictors of lymphatic spread, recurrence and survival are essential to guide surgical decision-making. Consistent with previous reports, we confirmed in our cohort a highly significant association between tumor size (pT stage) and ipsilateral nodal involvement, underlining the role of tumor size and depth of invasion (DOI) as a robust determinant of cervical metastasis pattern [47–49].

Our analysis revealed a significant correlation between pT stage and anatomical subsite. Anterior tumors were predominantly classified as early stage (pT1-2), whereas multifocal lesions involving both anterior and posterior regions were mostly advanced (pT2-3). Posterior tumors exhibited a more heterogeneous distribution but were still most often categorized as pT1-2. These findings align with previous reports indicating that tumor topography exerts a major influence on the pattern of nodal spread [50]. Posterior and multifocal lesions demonstrated a higher metastatic potential, which may be explained by their anatomical proximity of the tumor-node (T-N) tract to dense lymphatic networks in the posterior tongue and FOM [51, 52]. The interaction between tumor site and pN+ stage highlights the importance of integrating these parameters into risk stratification for elective neck management, with particular attention to the potential need for adjuvant treatment in those patients at higher risk of nodal disease [50].

Contralateral lymphatic dissemination remains a critical concern in TSCC. In our cohort, contralateral drainage was detected in 21.6% of patients, with a trend toward higher rates among those presenting with a greater ipsilateral nodal burden, whereas contralateral lymph node metastases were confirmed in 5.4%. Although statistical significance was not reached, this finding is consistent with previous reports associating contralateral drainage metastasis with advanced tumor stage and extensive ipsilateral nodal disease. These observations support that a high ipsilateral tumor load may compromise physiological compartmentalized lymphatic drainage barriers and facilitate crossover metastasis [4, 53]. Clinically, this underscores the importance of considering contralateral elective neck dissection in selected high-risk patients [54].

Analysis of recurrence patterns revealed moderate associations between advanced pT stage, higher nodal status and overall recurrence, encompassing local, regional and contralateral nodal events. Importantly, the absolute number of positive nodes did not predict recurrence, supporting previous evidence that biological features (e.g., PNI and ECE), as well as ratio-based metrics (e.g., lymph node ratio and log odds of positive nodes), could additionally precise locoregional control [43, 55].

The total number of metastatic lymph nodes was identified as a strong prognostic marker for survival. In line with recent evidence, each additional positive node was associated with an approximately 50% increase in mortality risk, underscoring nodal burden as a key predictor of survival in TSCC [43]. Clinically, this emphasizes the importance of precise quantification of nodal disease in guiding adjuvant treatment decisions and refining individual risk stratification.

Tumor size (pT stage) and advanced nodal stages were associated with poorer survival outcomes, in line with previous reports identifying tumor stage and nodal burden as key prognostic determinants in TSCC [56]. Moreover, PNI emerged as a significant predictor of inferior overall survival, corroborating recent evidence that PNI represents an independent adverse factor in TSCC [43, 57].

The strengths of this retrospective study include a clearly defined, single-entity TSCC cohort managed within standardized institutional pathways, comprehensive multimodal staging with [^18^F] FDG PET/CT and ultrasonography with robust histopathological confirmation. Detailed clinicopathological variables including lymphatic laterality, PNI, ECE and exact nodal counts were systematically captured and analyzed.

Several limitations of this study should be acknowledged. First, the retrospective, single-center design and modest sample size with particularly small subgroups limit the generalizability of our findings and may introduce selection bias. Overall, the descriptive analyses did not reveal statistically significant associations between mortality or recurrence and surgical neck management, primary tumor stage or nodal status in this cohort. The limited number of outcome events substantially reduced statistical power and precluded multivariable modeling. The principal conclusions of this study therefore rely on descriptive patterns. Univariate associations and clinically robust effects were observed, most notably the prognostic relevance of the total number of metastatic lymph nodes and the presence of perineural invasion (PNI), both of which remained consistent across analytic approaches. Furthermore, the relatively short median follow-up reflects the inclusion of recently treated patients. Consequently, survival outcomes should be interpreted with caution, as a substantial proportion of the cohort has not yet reached mature long-term follow-up. Second, by focusing on nodes deemed suspicious preoperatively, the dataset might overrepresent high SUV_max_ or large nodes, introducing spectrum and selection bias. Third, small-volume metastatic deposits remain a diagnostic blind spot for [^18^F] FDG PET/CT and likely contributed to reduced sensitivity. Fourth, depth of invasion (DOI) was not available for all patients, reflecting changes in histopathological reporting standards over the long inclusion period. Despite its guideline-anchored relevance, DOI could not be incorporated as a continuous predictor in this retrospective analysis. Because DOI is embedded within the pT category [40], its effect is partly captured in the postoperative pT-based analyses. Future prospective datasets with uniform DOI documentation are required to integrate DOI alongside imaging-based preoperative predictors.

Future studies should comprise large-scale, prospective, multicenter cohorts. Such datasets could enable for robust receiver operating characteristics (ROC) modelling and the derivation of clinically applicable cut-offs (e.g., SUV_max_, RECIST), while minimizing spectrum and selection bias. Hybrid imaging holds additional promise. [^18^F] FDG PET/MRI and multiparametric MRI with diffusion-weighted imaging may improve the detection of small-volume nodal disease in anatomically complex head and neck region. In addition, the evaluation of novel radiopharmaceuticals and lymphoscintigraphy might address false-positive/-negative trade-offs, especially in cN0 necks.

Methodologically, future studies should predefine robust endpoints (e.g., contralateral nodal failure, regional control, OS/RFS), use standardized acquisition and reporting protocols and incorporate external validation to assess model transportability. AI-assisted interpretation of radiologic findings and multimodal image fusion ([^18^F] FDG PET/CT ± MRI), integrated with established clinicopathologic predictors (e.g., pT/pN stage, PNI, ECE, DOI), might has the potential to enhance prognostic accuracy and further refine patient-specific treatment recommendations. Finally, beyond diagnostic accuracy, trials should prospectively capture patient-reported outcomes, functional morbidity (e.g., shoulder function, lymphedema) and cost-effectiveness, to ensure that imaging-guided, function-preserving neck management confers clinical and economic benefit in standard TSCC care.

## Conclusion

In conclusion, this study demonstrates that preoperative [^18^F] FDG PET/CT-detected nodal burden, tumor size and the presence of contralateral lymphatic drainage are potential predictors for patient-specific tailoring the extent and laterality of neck dissection in TSCC. Contralateral involvement was predominantly observed in lateral tongue carcinomas and showed a trend toward higher incidence with increasing ipsilateral nodal burden, highlighting its clinical relevance for surgical decision-making. In addition, univariate analysis indicated a trend toward higher recurrence rates in female patients, suggesting gender-related factors in risk stratification. These findings underscore the importance of integrating biological tumor behavior, sex-related differences and advanced imaging into patient-specific oncological strategies, refining the indication and extent of neck dissection beyond conventional staging. However, considering the outlined limitations, the presented data must be interpreted with caution and should be regarded as exploratory in nature and hypothesis-generating, requiring validation in prospective studies.

## Data Availability

No datasets were generated or analysed during the current study.

## Glossary

cTNM: Clinical Tumor, Node, Metastasis classification
DOI: Depth of invasion
ECE: Extracapsular extension
END: Elective neck dissection
FOM: Floor of mouth
NPV: Negative predictive value
OR: Odds ratio
OS: Overall survival
OSCC: Oral squamous cell carcinoma
PPV: Positive predictive value
RFS: Recurrence-free survival
RIS: Radiological information system
ROC: Receiver operating characteristics
SLNB: Sentinel lymph node biopsy
SOHND: Supraomohyoid neck dissection
STROBE: Strengthening the Reporting of Observational Studies in Epidemiology
SUV_max_: Standardized Uptake Value maximum
T-N tract: Tumor-node tract
TSCC: Tongue squamous cell carcinoma
VOI: Volume-of-interest
RECIST: Response Evaluation Criteria in Solid Tumors
